# Student progress decision-making in programmatic assessment: can we extrapolate from clinical decision-making and jury decision-making?

**DOI:** 10.1186/s12909-019-1583-1

**Published:** 2019-05-30

**Authors:** Mike Tweed, Tim Wilkinson

**Affiliations:** 1 Department of Medicine, University of Otago Wellington, Wellington, New Zealand; 2 University of Otago Christchurch, Christchurch, New Zealand

**Keywords:** Programmatic assessment, Decision-making, Policy

## Abstract

**Background:**

Despite much effort in the development of robustness of information provided by individual assessment events, there is less literature on the aggregation of this information to make progression decisions on individual students. With the development of programmatic assessment, aggregation of information from multiple sources is required, and needs to be completed in a robust manner. The issues raised by this progression decision-making have parallels with similar issues in clinical decision-making and jury decision-making.

**Main body:**

Clinical decision-making is used to draw parallels with progression decision-making, in particular the need to aggregate information and the considerations to be made when additional information is needed to make robust decisions. In clinical decision-making, diagnoses can be based on screening tests and diagnostic tests, and the balance of sensitivity and specificity can be applied to progression decision-making. There are risks and consequences associated with clinical decisions, and likewise with progression decisions.

Both clinical decision-making and progression decision-making can be tough. Tough and complex clinical decisions can be improved by making decisions as a group. The biases associated with decision-making can be amplified or attenuated by group processes, and have similar biases to those seen in clinical and progression decision-making.

Jury decision-making is an example of a group making high-stakes decisions when the correct answer is not known, much like progression decision panels. The leadership of both jury and progression panels is important for robust decision-making. Finally, the parallel between a jury’s leniency towards the defendant and the failure to fail phenomenon is considered.

**Conclusion:**

It is suggested that decisions should be made by appropriately selected decision-making panels; educational institutions should have policies, procedures, and practice documentation related to progression decision-making; panels and panellists should be provided with sufficient information; panels and panellists should work to optimise their information synthesis and reduce bias; panellists should reach decisions by consensus; and that the standard of proof should be that student competence needs to be demonstrated.

## Background

### The problem with decision-making in assessment

Much effort has been put into the robustness of data produced by individual assessments of students. There is an extensive literature on achieving robustness of assessment data at the individual test or assessment event level, such as score reliability, blueprinting, and standard setting [1–3]. This is especially so for numerical data [4], but increasingly also for text/narrative data [5]. However, decisions are more often made by considering a body of evidence from several assessment events. This is increasingly the case as a more programmatic approach to assessment is taken [6]. For example, the decision on passing a year is becoming less about a decision on passing an end of year examination and more about a decision based on synthesising assessment results from across an entire year. Despite these changes, there is a gap regarding the pitfalls and ways to improve the aggregation of information from multiple and disparate individual assessments in order to produce robust decisions on individual students [7].

In this paper we draw parallels between student progression decision-making and clinical decision-making, and then within the context of decisions a made by groups, we will draw parallels between progression decision-making and decision-making by juries. Finally, exploration of these parallels leads to suggested practical points for policy, practice and procedure with regard to progression decision-making. There are many examples of decision-making that could be used but we chose clinical decision-making as it is familiar to healthcare education institutions, and jury decision-making as it is a relevant example of how groups weigh evidence to make high-stakes decisions.

### Progression decision-making: parallels in clinical decision-making

The decision-making around whether a student is ready to progress (pass) or not (fail) has many parallels with patient diagnosis [8]. For both assessment progression decisions and patient diagnosis decisions, several pieces of information (a mix of numerical and narrative/text with varying degrees of robustness), need to weighed up and synthesised. Patient diagnosis decisions and subsequent decisions on management can be high-stakes in terms of impact on the patient and/or healthcare institution. Likewise progression decisions and the consequences carry high-stakes for students, educational institutions, healthcare institutions, patients, and society.

#### Aggregating information to make decisions

Clinicians and clinical teams combine various pieces of information efficiently and accurately using heuristics [9–14], however clinical decision-making regarding patient diagnoses can be prone to biases and inaccuracies [12, 15–18]. Just as metacognitive awareness of such biases and errors [15, 16] is postulated to lead to improved clinical decision-making [19–21], we suggest that an awareness of such biases in combining assessment information, and ways to address this, could also improve the robustness of progression decisions.

#### Gathering information

In the clinical setting, data used to inform the decision-making of a patient diagnosis may come from the consultation and associated investigations. The history is almost entirely narrative/text, the clinical exam is mostly narrative/text with some numerical data, and investigations are a mixture of narrative/text and numerical data. Clinical decision-making leading to a diagnosis can be quick and efficient [15], but sometimes it is more difficult and the clinician may need to obtain more information, weigh up different options, and/or weigh up conflicting pieces of evidence.

The process of obtaining additional information may include repeating data collection, e.g. revisiting the consultation and investigations; approaching the issue from a different perspective, e.g. obtaining a computerised tomography scan to complement a plain radiograph; and/or looking for an entirely new and different source of information, e.g. getting a biopsy [15]. The nature of this additional information will depend on the information obtained so far, as doing the same extra tests on all patients regardless of what is already known is not good clinical practice. Consideration is also given to the most appropriate investigations in terms of efficiency, risk/benefit, and cost [22, 23], to answer the clinical question posed.

In clinical decision-making it is inefficient, and sometimes harmful, to keep collecting data or undertaking investigations once a diagnosis is secure. There are parallels with this, in terms of progression decision-making: obtaining additional information to inform progression decision-making may include sequential testing, whereby testing ceases for an individual student when sufficient information has been gathered [24]. This could be extrapolated to programmes of assessment whereby assessments cease when sufficient information is available on which to base a progress decision. The stakes of the decision would inform the strength and weight of the information required for a sufficiency of information. Just as for clinical decision-making, more of the same type of assessment may not improve progress decision-making, and a new perspective or an entirely new data source may be required. Instead of asking a student to repeat an assessment, a period of targeted observation, closer supervision or different assessments might be preferable to provide the required sufficiency of information. The nature of the extra information required will depend on what is already known about the individual, and may vary between students. The resulting variable assessment may generate concerns over fairness. In response, we would argue that fairness applies more to the robustness and defensibility of the progression decision, than to whether all students have been assessed identically.

#### Aggregating conflicting information

In clinical decision-making it is often necessary to weigh up conflicting pieces of evidence. Information gathered from history, examination, and investigations might, if considered in isolation, generate different lists of most likely diagnoses, each of which is held with uncertainty. However, when all the information is synthesised, the list of most likely diagnoses becomes clearer, and is held with increasing certainty [25]. Likewise in progression decision-making, considering single pieces of information generated from independent assessment events might generate different interpretations of a student’s readiness to progress, but when these single pieces are synthesised, a more robust picture is constructed.

Synthesising data from multiple sources is possible for healthcare policy makers and practitioners [26–28]. Some data synthesis is done better mechanically or by algorithms than by individual clinicians [29], but better results may be achieved if fast and frugal heuristics are combined with actuarial methods [30]. In progression decision-making, combining scores using algorithms is possible [31], but equally plausible algorithms can lead to different outcomes [32, 33]. It may be easy simply to add test results together, but the result may not necessarily contribute the best information for decision-making purposes [31].

For clinical decision-making, strategies to improve decision-making include consideration of the health systems, including the availability of diagnostic decision support; second opinions; and audit [12]. A lack of checking and safeguards can contribute to errors [34]. Extrapolating this to progression decision-making, all assessment results should be considered in context, and decision support and decision review processes used.

#### Screening tests and diagnostic tests

Testing for disease in clinical practice can include a screening programme which requires combining tests, such as a screening test followed by a confirmatory test [35]. This can be extrapolated to progression decision-making [8], especially when data are sparse [36]. Generally, decision-making from clinical tests and educational assessments has to balance the sensitivity with the specificity of a test to help inform the decision. This is influenced by the purpose of the individual assessment and by the purpose of the assessment testing programme [8]. A screening programme for a disease will generally have a lower specificity and higher sensitivity, and a confirmatory test a lower sensitivity and higher specificity [35]; the predictive value of the test will be dependent on disease prevalence. Hence despite apparently excellent sensitivity and specificity, if the prevalence is very high or low, a testing programme can be non-contributory, or worse still, potentially harmful [8]. Such biases associated with educational assessment are discussed later.

#### Risks associated with decisions

The consequence and risk of incorrect clinical decisions, or deviation from optimal practice, can vary significantly from no clinically significant consequence to fatality [37]. Adverse consequences and risks occur even with optimal practice. Drugs have side effects, even when used appropriately, and sometimes these risks only come to light in clinical practice [38].

Healthcare educational institutions have a duty of care to take the interests of both students [39] and society [40] into account when making progression decisions on students. This dilemma of making decisions for individuals which have an impact not only on that individual, but also society, is explored further in the section on jury decision-making.

#### When the decisions get tough

Some decisions are made more difficult by the context, such as time-pressured decision-making in clinical practice [41] and high-stakes decision-making [42]. Even when correct answers are known, time-pressure increases uncertainty and inaccuracy in decision-making. It is important that educational institutions provide decision-makers with sufficient time to make robust decisions.

In addition, there are some questions that are impossible for an individual to resolve [34]. The diagnosis may not be straightforward because decisions may have significant consequences, and multiple specialised pieces of information or perspectives may need to be combined in order to advise optimal care. In these circumstances a second opinion may be requested [12]. Increasing the number of people considering the available data can be a better method than increasing the available data where this is not practical or safe. Multi-disciplinary teams, multi-disciplinary meetings, and case conferences can enhance patient care by using multiple people help to make decisions on aggregated information. In certain situations such group decision-making improves outcomes for patients [43].

One of the highest-stakes progression decisions on healthcare professional students is at graduation. The institution needs to recommend to a regulatory authority, and thereby society, that an individual is ready to enter the healthcare profession, and will be at least a minimally competent and safe practitioner. Given the potential high-stakes and complexity of the information to be considered, a panel is often part of decision-making in programmatic assessment [6]. The panellists bring different perspectives, and the longstanding assertion is that the collective is better than the component individuals [44].

### Comparing decision-making by individuals and groups

When aggregating information, the average of many individuals’ estimates can be close to reality, even when those individual estimates may be varied and lie far from it [44, 45]. This ‘wisdom of the crowd’ effect may not be true in all situations. When people work collectively rather than individually, this effect may be less apparent, as social interactions and perceived power differentials within groupings influence individual estimates. The resulting consensus produced is no more accurate, yet group members may perceive that they are making better estimates [45]. Further, the use of average, whether mean or median, to demonstrate this effect reflects the strength of how this effect works for numerical rather than narrative data, it is a mathematical effect [45]. The apparent reassurance that groups make better decisions than individuals may be misplaced when it comes to narrative data or collective decisions, unless precautions are taken.

Errors in decision-making can arise due to faults in knowledge, data gathering, information processing, and/or verification [46]. There are biases and errors in individual’s decision-making [10, 12, 15, 17, 18, 47], some of which are also evident in group decision-making [48–50]. In comparing biases and errors in decisions made by individuals with those made by groups, some are attenuated, some amplified, and some reproduced, with no consistent pattern by categorisation [48]. These biases and errors, as they relate to individual and group progression decision-making, are shown in Table 1.

**Table 1:** Descriptions from clinical and progression decision-making, where individual and group decisions have been compared

Explanation of type of bias and/or error [48]	Description and example from clinical decision-making	Description and example from progression decision-making	Effect on decision-making as a group vs as an individual [48]
Framing: decisions vary with the context in which the information is presented.	A patient has had several visits to an ED with a headache, and on each occasion has been diagnosed as having migraine. On this visit, the clinician assumes that the patient has migraine again [15].	Substandard performance at the start of year creates the label of a “poor performing” student, and performance later in year is assumed to represent poor performance [51]. A similar bias for above-standard performance is also possible.	Mixed effects as amplification, attenuation and no change have all been reported
Preference reversal: the judgement outcome depends on how the data are presented	Choices made by clinicians and patients when considering management options will vary dependant on information how information is presented [52]. As an example, the probability of getting a condition whilst taking a drug is 96%, and when not taking the drug is 98%. Put another way, the chance of getting the disease is halved (from 4 to 2%). The prevalence of side effects form the drug is 10%: put another way 90% have no problems at all. The patient decision may alter depending on which figures are provided.	When faced with making a decision for a student at the borderline of satisfactory performance, the outcome may differ if the focus is on being fair to the student or if the focus is on protecting the public.	Mixed: amplified on and attenuated
Theory-perseverance effect: confirmation bias (only looking for information that will support a decision) and ascertainment bias (only finding information that will support a decision).	Confirmation bias occurs when a clinician only seeks out evidence that supports a the proposed diagnosis, such as only asking about cardiac symptoms in a person with breathlessness [15]. Ascertainment bias occurs when a clinician preferentially finds supporting evidence, such as finding evidence of heart failure in a patient with breathlessness who has been noncompliant with diuretic medication [15].	When observing a student, the examiner forms an initial first impression of the student as being below standard, and thereafter only looks for evidence of poor performance/only finds evidence of poor performance. A similar bias for above-standard performance can also occur [53].	Attenuated by group
Weighting sunk costs: continue to invest in a losing transaction because of losses already incurred.	A decision to continue ineffective treatment, such as ongoing treatment for progressive malignancy [54].	A student who has progressed a significant way through a course (e.g. to final year), before their substandard performance comes to attention. They may be harder to fail as “they’ve got this far”, and be given the benefit of the doubt that they are a potentially failing student [36, 55].	Amplified by groups
Extra-evidentiary bias: Irrelevant information influences decision-making.	Clinical decision-making regarding an individual patient may be informed by trial results, which then requires extrapolation by clinicians identifying similarities and differences between the patients in the trial and the individual patient. This process is often influenced by extra-evidentiary considerations, such as personal clinical experience [56]. Additionally, combining information as part of clinical decision-making requires appropriate aggregation rules utilising the tools of mathematics (e.g. set theory, symbolic logic, and Boolean algebra) to support more reliable decisions [56].	A body of assessment evidence suggests a student is passing, but an influential senior staff member provides a single anecdote of substandard performance that sways the decision. This can work both in favour of, and against the student [57].	More amplification than attenuation for groups
Hindsight bias: knowing the outcome alters recollections; assigning inferences; ignoring prevalent circumstances.	When events are viewed in hindsight, there is a strong tendency to attach a coherence, causality, and deterministic logic to them, such that no other outcome could possibly have occurred, thereby distorting the perception of previous decision-making [15].	Most doctors with professional conduct problems in practice had professional conduct problems in medical school [58, 59]. Awareness of this could lead a medical school to erroneously fail any student with professional conduct problems during a medical course, yet the vast majority of students with professional conduct problems become clinicians with no professional conduct lapses.	Attenuated for groups
Insensitivity to base rate (underuse of representative heuristic): frequency within population is ignored in estimating probability	If all causes of pleuritic chest pain are considered to have equal pre-test probabilities, then they are all assumed to have equal prevalence rates. This can lead to over-investigation of less likely causes (e.g. pulmonary embolus) and therefore an overestimation of the post-test likelihood [15].	A single performance just below the standard (e.g. in an end-of-year OSCE) in an assessment with a high pass rate (e.g. > 95%) by a student is given too much weight, when the student has clearly been above standard to date in all equivalent assessments, and the pre-test probability of passing should be high [60].	Mixed results
Overuse of presentative heuristic: overreliance on some salient information; stereotyping based on similarities.	The patient’s symptoms and signs are matched against the clinician’s mental templates for their representativeness. Clinicians base diagnostic decisions about whether or not something belongs to a particular category by how well it matches the characteristics of members of that category [15]. A patient presenting with atypical symptoms and signs, such as a young female with a history of psychiatric disease, can lead to myocardial infarction not being considered, and the patient being sent home from Emergency Department.	A student who is a member of a specific group (e.g. male ethnic minority) that performs less well in assessments [61] is expected to demonstrate lower performance.	Mixed: Amplified by groups or no effect
Overconfidence (miscalibration): belief in the probability of being correct is greater than actual.	Overconfidence by a clinician thinking they know more than they do, leading to gathering insufficient information [15].	A clinician may consider themselves, rightly or wrongly, to be a good clinician, and therefore also assumes they will also be a good assessor. An individual will put greater weight on their decisions than is justified by the evidence [62].	Mixed effects

Groups, like individuals, undertake several processes in coming to a decision. The process of individuals gathering into a group can influence information recall and handling [48]. Although there is a significantly greater literature on individuals making decisions, groups making decisions can also be prone to biases [63] and this can arise from many sources [43]. In the context of progression decision-making, a group’s initial preferences can persist despite available or subsequently disclosed information [64], a bias similar to premature closure in diagnostic decision-making [15]. Group members may be aware of interpersonal relationships within the decision group, such as the undue weight of a dominant personality, and these perceptions can influence an individual’s contribution and discussion of information [48]. Persuasion and influence occur during discussion of a candidate assessment. Outliers who initially score candidates higher are more likely to reduce their score, while outliers who initially score the candidates lower are less likely to increase their score, with the result that consensus discussion is likely to lower candidate scores and therefore reduce the pass rate [65].

### A jury as an example of high-stakes decision-making by a group

Jury decision-making is an example of a group making a high-stakes decision [48], that has been extensively researched and therefore could offer insights into progression decision-making. There is significant literature on decision-making, biases, and errors by jurors and/or juries [49, 50, 66–76], including a summarising review [77]. There are similarities between the main purpose of the group of jurors considering all the evidence (with the aim of reaching a high-stakes verdict which is often a dichotomous guilty or not guilty verdict) and the main purpose of a group of decision-makers to consider all the assessment data (with the aim of reaching a high-stakes verdict of pass or fail). Jury decision-making, like progression decision-making, but unlike other group decision-making described, does not address a problem with a known correct answer [48, 66].

The relative contribution to the decision brought about by jurors and juries varies with the task [50]. As for clinical decision-making, there are heuristics which can improve the accuracy and efficiency of decisions, but when these produce less accurate or less efficient results, they are seen as biases. Susceptibility to variation and bias has been reported for simulated jurors and/or for some real juries, with factors that include [49, 50, 66–77]:

- Defendant and/or victim/plaintiff factors. This includes personal factors such as gender, race, physical appearance, economic background, personality, injuries, pre-trial publicity, disclosure of defendants prior record, freedom from self-incrimination, being individual or corporation, courtroom behaviour;
- Juror factors. This includes authoritarianism, proneness to be pro-conviction or pro-acquittal, age, gender, race, social background, recall of evidence, understanding of evidence, ignoring information as instructed, prior juror experience;
- Representative factors. This includes legal representation factors such as gender, written/verbal representation, clarity, style and efficiency of presentation;
- Evidence factors. This includes imagery of evidence (the more visual or more visually imaginable), order of presentation, nature of evidence;
- Crime factors. This includes the severity or type of crime;
- Judge factors. This includes the content of the instructions or guidance given;
- Jury membership factors. This includes the mix of aspects such as social background mix, racial mix.

There are similarities in some of these factors in relation to progression decision-making. The ease of building a story influences both the decisions and the certainty in those decisions [71], akin to the availability bias. The juror bias due to initial impression [67, 75, 77] is akin to anchoring. People may identify with similar people; a “people like us” effect may be present [78]. For progression decision-making some of these effects can be mitigated by anonymisation of students, as far as possible.

One difference between a jury and a panel making a progression decision, is that a juror does not provide information to their co-jurors. In contrast, a member of a progression decision panel might also have observed the student and can provide information. Lack of observation by the decision-makers can be a benefit in decision-making, as it removes a potential source of bias: a single anecdote can inappropriately contradict a robust body of evidence [57]. Additionally, bias produced by incorrect evidential recall is less of an issue than evidence presented to the panel for deliberation.

The programmatic assessment panel may be closer to a Supreme Court panel of judges rather than a jury of lay-people and peers, but there is little research on the decision-making and deliberations of panels of Supreme Court judges, which are conducted in closed-door meetings.

#### Jury decision-making style

Jury deliberation styles have been shown to be either evidence-driven, with pooling of information, or verdict-driven, which start with a verdict vote [68]. Evidence-driven deliberations take longer and lead to more consensus; verdict-driven deliberations tend to bring out opposing views in an adversarial way. When evidence-driven deliberations lead to a significant change of opinion, it is more likely to be related to a discussion of judge’s instructions [68]. If the decision rules allow a majority vote verdict without consensus, a small but real effect is seen [77]: juries will stop deliberating once the required quorum is reached. Verdict voting can be subject to additional biases such as voting order where people alter their vote depending on the votes given to that point [77]. Group discussions are not without potential problems, in that they can generate extreme (more honest) positions. Ninety percent of all jury verdicts are in the direction of the first ballot majority [66], but a small and not insignificant number are swayed by deliberation. Once individuals state their individual decisions and rationales, diffusion of responsibility within a group may lead to riskier opinions being stated, and therefore riskier decisions being made [66].

Extrapolating this to the context of progression decision-making, an optimal approach is consensus decisions that are based on evidence, whilst attending to the rules and implementation of policy and process.

#### Jury leadership

Based on what we know about jury decision-making processes, the jury foreperson, the equivalent of the assessment progress panel chair, needs the skills to preserve open discourse, whilst maintaining good process in decision-making. The jury foreperson can be influential [77], and individual jurors can hold extreme views, though the process of jury selection usually mitigates against the selection of people with extreme views [66].

In choosing progress decision-makers, consideration should be given to the skills that are required to make high-stakes decisions based on aggregating information, rather than skills and knowledge relating to clinical practice.

#### Jury leniency and failure to fail

Is there a parallel between leniency towards the defendant and the failure to fail phenomenon [55]? Juries are instructed to presume innocence [67]: if one is to err in a verdict, leniency is preferred [79]. Legal decision-making has two components: the probability of supporting a decision, and threshold required to support that decision [66]. It is possible to support a decision but still retain a degree of doubt. The effect of standard of proof (reasonable doubt) required on juror and jury outcomes is significant [69, 77]. If in doubt, a jury will favour acquittal [48, 63]. Jury deliberations tend towards leniency [72, 75], with most leniency is accounted for by the requirement of standard of proof [72].

A similar effect has been observed in progression decision-making where, if in doubt, the decision is usually to pass the student [55]. The onus is on the jury to presume innocence unless finding guilt proven, but is the onus on the progress panel to find student competent proven? Too often this onus is erroneously misinterpreted as presuming competence unless finding incompetence proven. This can manifest as a discounting of multiple small pieces of evidence suggesting that competence has not yet been demonstrated [36].

### Suggestions to attend to in order to promote robustness of decisions made relating to student progression

We now propose some good practice tips and principles that could be used by progression decision-makers. These are based on the previously outlined evidence from clinical decision-making and jury decision-making, and from additional relevant literature.

#### Educational institutions, decision-making panels, and panellists should be aware of the potential for bias and error in progression decisions

Being consciously aware of the possibility of bias is the first step to mitigate against it [19–21]. Such biases can occur both for individuals making decisions and for groups making decisions. Extrapolating from clinical decision-making, the challenge is raising awareness of the possibility of error by decision-makers [12]. Clinicians failing to recognise and disclose uncertainty in clinical decision-making is a significant problem [47, 80]. However, even when there is uncertainty over student performance, decision panels still need to make a decision.

#### Decisions should be made by appropriately selected decision-making panels

Extrapolating from clinical decision-making, strategies to improve individual decision-making include promotion of expertise and metacognitive practice. A lack of expertise can contribute to errors [34], hence panel members should be selected with appropriate expertise in student outcome decision-making, rather than assessment content, and reflections on decision quality should include quality assurance in the way of feedback on decisions and training for decision-making. As such, the panel should be chosen on the basis of its ability to show metacognition in recognising bias, rather than status/seniority, familiarity with assessment content, or familiarity with the students.

Even a panel of experienced decision-makers is not without the potential for bias [81], but there are possible solutions that can be implemented at the policy, procedure and practice levels. Given the potential for professional and social interactions between students and staff, there should be policy, procedure, and practice documentation for potential conflicts of interest. If a decision-maker is conflicted for one or more students, then they should withdraw from decision-making. Potential conflicts of interest are far more likely to relate to individual decision-makers and individual students, and should be dealt with on a case-by-case basis guided by an appropriate policy. Examples of conflict might include more obvious relationships with family members, but also with mentors/mentees and those with a welfare role with students.

#### Educational institutions should have publicly available policies, procedures, and practice documentation related to assessment events and the associated decision-making

Improving jury performance can be achieved through improving procedural issues [77]. These include, but are not necessarily limited to, the following: a thorough review of the facts in evidence, accurate jury-level comprehension of the judge’s instructions, active participation by all jurors, resolution of differences through discussion as opposed to normative pressure, and systematic matching of case facts to the requirements for the various verdict options. Likewise, from the perspective of a progression panel decision, these would equate to: a thorough review of the information provided, accurate comprehension of the policy, active participation by all panel members, resolution of differences through discussion and consensus, and systematic matching of information to the requirements for the assessment purpose and outcomes. While some might argue that these components are already implicit in many decision-making processes, the quality of decision-making may be improved if such components are made more explicit.

#### Panels and panellists should be provided with sufficient information for the decision required

Group discussions can improve recall of information [48], and some of the benefit of juries, as opposed to jurors, relates to improved recall by a group compared to individuals [66, 67, 74]. Multiple jurors produce less complete but more accurate reports than individual jurors [66].

In progression decision-making, it is unlikely that panellists will have to rely on recall for specifics of information or policy when making decisions, but the panel will need to decide if they have sufficient information (quality and quantity) in order to reach a decision for an individual student. Where there is insufficient information, but more may become available, this should be specifically sought [36], and a decision deferred. Where further information will not become available, the question should then turn to where the onus of the burden of proof lies.

#### Panels and panellists should work to optimise their information synthesis and reduce bias

The act of deliberation and discussion within groups attenuates many of the biases and errors of individuals [48], as outlined in Table 1. Some biases, such as extra-evidentiary bias, can be amplified in group decision-making, an example being where provision of an anecdote could unduly influence a group’s decision [57].

Progression decision-making requires consideration of all information and the context, with decision support and decision review. External review might extend beyond just reviewing the decisions, to an external review of the underlying panel process, procedures, and practices. Not every panel discussion needs external review, but policy review associated with regular external observation would be appropriate.

#### Panellists should reach decisions by consensus

Consensus decision-making rather than voting avoids adversarial decision-making. In an attempt to produce fairness within a courtroom, facts are uncovered and presented in an adversarial manner, with information being questioned by opposing legal representation [67]. This results in the appearance of evidential unreliability and contentiousness. Similarly, when faced with information presented in an adversarial way, progression decision-making panels might view the information as being less reliable, and therefore insufficient to make a robust decision.

#### The burden of proof should lie with a proven demonstration of competence

For high-stakes pass/fail decision-making, the standard of proof should be proof that the student’s competence is at a satisfactory standard to progress. The assumption is often that the student is competent, until proved otherwise. In contrast to “innocent until proven guilty”, we suggest students should be regarded as incompetent until proven competent, reflecting the duty for healthcare educational institutions to protect society [40].

The predictive value of a test result is affected by the pre-test probability or prevalence, even though sensitivity and specificity may not change. This pre-test probability or prevalence of passing should increase as a cohort progresses through the course, as less able students are removed. Therefore, incorrect pass/fail decisions are relatively more likely to be false fails (true passes) than false passes (true fails), and when an assessment is equivocal, it is more likely that the student is satisfactory than not. However, as a student progresses through the course and the opportunities for further assessment are reduced. As graduation nears, the stakes and impact of an incorrect pass/fail decision increases. Although pre-test probability or prevalence considerations would favour passing the student, the duty of the institution to meet the needs and expectations of society should override this.

## Conclusion

We provide a call for metacognition in progression decision–making. We should be mindful of the strengths of combining several pieces of information to construct an accurate picture of a student, but should also be mindful of the sources of bias in making decisions. While we acknowledge that many institutions may already be demonstrating good practice, awareness of biases and the suggested process outlined in this paper can serve as part of a quality assurance checklist to ensure hidden biases and decision-making errors are minimised. Drawing on one’s experience of clinical decision-making and an understanding of jury decision-making can assist in this.
